# Association of Sarcopenia and Lower Bone Density With Positional Vertigo in the Morning: Insights From a Nationwide Survey

**DOI:** 10.1002/jcsm.70219

**Published:** 2026-02-03

**Authors:** Eun Ji Kim, Eunjin Kwon, Sukyoung Jung, Ji‐Soo Kim, Seong‐Hae Jeong

**Affiliations:** ^1^ Department of Neurology Chungnam National University Hospital Daejeon Republic of Korea; ^2^ Department of Neurology Chungnam National University School of Medicine Daejeon Republic of Korea; ^3^ Department of Healthcare Policy Research Korea Institute for Health and Social Affairs Sejong Republic of Korea; ^4^ Department of Neurology Seoul National University College of Medicine Seoul Republic of Korea; ^5^ Department of Neurology Dizziness Center and Clinical Neuroscience Center Seoul National University Bundang Hospital Seongnam Republic of Korea

**Keywords:** body composition, bone mineral density, postural balance, sarcopenia, vertigo, vestibular system

## Abstract

**Background:**

This study aimed to identify associations of sarcopenia, obesity and low bone mineral density (BMD) with morning positional vertigo (PV) and to examine whether these associations differ according to vestibular function status in a nationally representative sample of Korean adults.

**Methods:**

We analysed data from 8512 adults aged ≥ 40 years (50.03% women, mean ± standard error [SE] = 54.06 ± 0.19) who participated in the Korean National Health and Nutrition Examination Survey 2008–2010. Morning PV was defined as severe vertigo when turning in bed or rising in the morning within the past year. Vestibular impairment was assessed using the modified Romberg test (eyes closed, standing on a compliant foam surface). Participants were classified into three groups: controls (no dizziness), morning PV with normal Romberg test results and morning PV with abnormal Romberg test results (suggesting vestibular dysfunction). Body composition—including appendicular skeletal muscle mass, fat mass and BMD—was measured. Sarcopenia was defined according to the Asian Working Group for Sarcopenia 2019 criteria, and abnormal BMD was defined as T‐score < 1.0. Weighted multinomial logistic regression analyses were adjusted for age, sex, income, comorbidities, lifestyle and psychosocial variables.

**Results:**

The weighted 1‐year prevalence of morning PV was 12.58% (95% confidence interval [CI], 11.38–13.89). Abnormal Romberg performance, indicating vestibular dysfunction, was present in 0.96% of all survey respondents and in 7.66% of those with morning PV. Participants with vestibular‐impaired morning PV were older (mean age ± SE = 66.47 ± 1.49), predominantly women, and had higher rates of sarcopenia (38.33%) and low BMD (86.39%) than controls. In adjusted models, sarcopenia independently predicted vestibular‐impaired morning PV (odds ratio [OR], 1.94; 95% CI, 1.14–3.29; *p* = 0.014). Sensitivity analysis restricted to current morning PV confirmed the association between sarcopenia and vestibular impairment (OR, 2.97; 95% CI, 1.08–8.12; *p* = 0.035) and demonstrated that lower BMD (minimum T‐score) was inversely associated with vestibular dysfunction (OR, 0.49; 95% CI, 0.25–0.99; *p* = 0.046).

**Conclusions:**

Morning PV is common among middle‐aged and older adults and is associated with systemic frailty markers, particularly sarcopenia and bone loss, in the presence of vestibular dysfunction. These associations were more pronounced in older adults, suggesting a vestibulo‐musculoskeletal interaction that may contribute to balance impairment and functional decline with aging. Screening for sarcopenia and bone health, along with vestibular and lifestyle interventions, may help reduce recurrent vertigo and improve functional aging.

## 1 Introduction

Dizziness is a frequent cause of outpatient and emergency visits [1], but diagnostic evaluation remains challenging because of overlapping symptom profiles and limited vestibular expertise, often leading to delayed or inconsistent care [2]. Because the vestibular system plays a central role in balance [3], clinical assessments have traditionally focused on vestibular function; however, musculoskeletal and metabolic factors have often been overlooked, despite their important and potentially modifiable contributions to postural stability [4, 5].

In clinical neurotology, contemporary diagnostic approaches increasingly emphasize a timing‐and‐triggers framework to characterize dizziness based on when symptoms occur and what provokes them [6]. Morning positional vertigo (PV), defined as vertigo elicited shortly after waking with head movement, represents a symptom‐defined subtype that aligns well with this model. Large population‐based surveys, such as the National Health and Nutrition Examination Survey (NHANES) and the Korean NHANES (KNHANES), have provided important insights into dizziness, balance impairment and systemic health indicators [7, 8]. However, prior analyses have typically treated dizziness as a single entity and have not examined morning PV as a distinct phenotype.

Recent evidence suggests that body composition may influence vestibular symptom expression and compensatory mechanisms. Studies have reported isolated associations between lower bone mineral density (BMD), sarcopenia, and obesity and benign paroxysmal PV (BPPV) [9, 10], reduced muscle mass and chronic dizziness [11], and elevated visceral adiposity with concomitant muscle loss in unilateral vestibular hypofunction [12]. However, these components have generally been evaluated independently. Integrated analyses linking body composition to dizziness have only recently emerged. In a nationwide study, we demonstrated that sarcopenia was significantly associated with dizziness accompanied by vestibular dysfunction, whereas no such association was observed in dizziness without vestibular impairment [13]. These findings suggest that the relationship between body composition and dizziness may depend on vestibular function status. Whether this pattern extends to morning PV remains unknown.

To date, no epidemiologic study has examined morning PV in relation to integrated body‐composition measures. Therefore, using nationally representative data, this study investigates the associations of musculoskeletal, metabolic and psychosocial factors with morning PV, with particular emphasis on effect modification by vestibular function. By evaluating muscle, bone and fat simultaneously, this study addresses an important gap in the literature and offers new insight into the systemic contributors to morning PV.

## 2 Methods

### 2.1 Study Population

**FIGURE 1:**
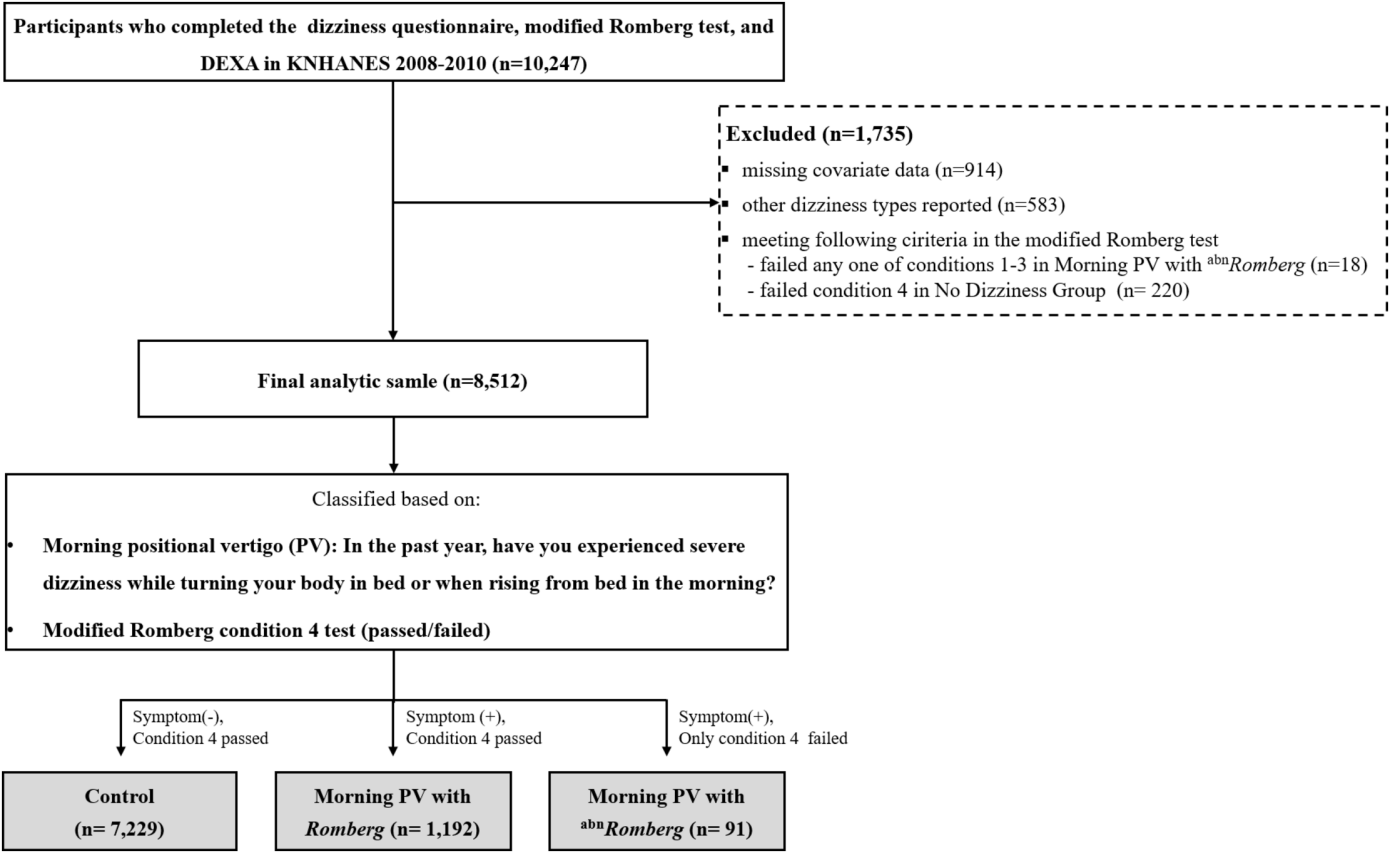
Flowchart illustrating participant selection and group classification. Participants who completed the dizziness questionnaire, modified Romberg test and DEXA components of the KNHANES 2008–2010 were identified (*n* = 10 247). Exclusion criteria were missing covariate data (*n* = 914), dizziness types other than morning PV (*n* = 583) and failure to meet specific modified Romberg test criteria (*n* = 238), leaving a final analytic sample of 8512 participants. Morning PV was identified by a ‘yes’ response to the question, ‘Do you experience vertigo when turning in bed or upon rising?’ Based on PV symptoms and performance on Condition 4 of the modified Romberg test (eyes closed, standing on foam), participants were classified into three groups: morning PV with normal *Romberg* test results (symptom + passed Condition 4; *n* = 1192), morning PV with ^abn^ *Romberg* test results (symptom + failed Condition 4; *n* = 91) and a control group (no symptom + passed Condition 4; *n* = 7229). abn, abnormal; DEXA, dual‐energy X‐ray absorptiometry; KNHANES, Korean National Health and Nutrition Examination Survey; PV, positional vertigo.

This cross‐sectional study used data from the 2008–2010 KNHANES, a nationally representative survey conducted by the Korea Centers for Disease Control and Prevention. KNHANES employs a complex, stratified, multistage probability sampling design and integrates three components: a health interview, a health examination and a nutrition survey. All components are conducted at designated health examination centres by trained personnel under standardized protocols. During the 2009–2010 cycles, participants aged ≥ 40 years completed a dizziness questionnaire, underwent the modified Romberg test and received whole‐body dual‐energy X‐ray absorptiometry (DEXA) scans [14]. Self‐reported dizziness symptoms in KNHANES are collected through structured questionnaire items that have been widely used in national epidemiologic research. Similar symptom‐based assessments of vestibular vertigo and PV have demonstrated good diagnostic validity in population settings, with structured interviews showing high sensitivity and specificity for vestibular vertigo compared with neurotologic evaluations in prior validation studies [15]. This framework supports the use of symptom‐based definitions for identifying PV in large‐scale surveys, although these classifications represent epidemiologic—not diagnostic—entities. Of the initial 10 247 participants, 1735 were excluded because of missing covariates (*n* = 914), other types of dizziness (*n* = 583) or failure to meet the relevant Romberg test criteria (*n* = 238). Specifically, exclusions included participants who failed to meet Conditions 1–3 in the morning PV (*n* = 18) or those who reported no dizziness but failed to meet condition (*n* = 220) (Figure 1).

Participants were classified based on two criteria:

1. A ‘yes’ response to the question, ‘In the past year, have you experienced severe vertigo while turning in bed or rising in the morning?’ Participants who also indicated that they still had the condition were classified as having *current morning PV*, indicating an ongoing symptomatic state.
2. Performance on the postural stability test corresponding to Condition 4 of the modified Romberg test. This condition requires participants to stand with their eyes closed on a compliant foam surface. Inability to maintain balance for ≥ 20 s under conditions of reduced visual and proprioceptive input was interpreted as vestibular dysfunction (^*abn*^ *Romberg*) [7, 8, 16, 17]. Details of this test have been described previously [13].

Group classification

Based on the presence of morning PV and performance on the modified Romberg test, participants were categorized into three groups [14]:

1. Control group (*n* = 7229): Participants who reported no dizziness or balance issues in the past year and passed Condition 4 of the modified Romberg test, indicating intact vestibular function
2. Morning PV with *Romberg* group (*n* = 1192): Participants who reported morning PV but passed Condition 4 of the modified Romberg test, indicating preserved vestibular function
3. Morning PV with ^*abn*^ *Romberg* group (*n* = 91): Participants who reported morning PV and failed Condition 4 of the modified Romberg test, suggesting vestibular dysfunction

### 2.2 Body Composition Analysis

Body composition—including BMD, skeletal muscle mass and body fat percentage—was measured using a DEXA Discovery‐W fan‐beam densitometer (Hologic Inc., Marlborough, MA, USA).

Sarcopenia was defined according to the Asian Working Group for Sarcopenia (AWGS) 2019 criteria as an appendicular skeletal muscle mass index < 7.0 kg/m^2^ in men and < 5.4 kg/m^2^ in women [18]. These thresholds were specifically developed for Asian populations and are considered the recommended diagnostic standard for Korean adults. Obesity was defined as a body fat percentage ≥ 25% in men or ≥ 35% in women. These cutoffs are consistent with the American Association of Clinical Endocrinologists (AACE)/American College of Endocrinology guidelines and are used in Korean national surveillance (e.g., the 2024 Obesity Fact Sheet), reflecting evidence that East Asian populations exhibit increased cardiometabolic risk at these thresholds [19]. BMD was evaluated using T‐scores at the total femur, femoral neck and lumbar spine. The minimum (min) T‐score was defined as the lowest value among these three sites.

Osteopenia was defined as a min T‐score between −2.5 and −1.0, and osteoporosis was defined as a T‐score ≤ −2.5. These diagnostic criteria follow the World Health Organization (WHO) definition and represent the internationally accepted standard routinely applied in Korean epidemiologic and clinical practice. In this study, abnormal BMD was defined as a min T‐score < −1.0, thereby encompassing both osteopenia and osteoporosis [20].

### 2.3 Risk Factors and Covariates

The demographic covariates included in our analysis were age, sex, body mass index (BMI) and household income. Clinical covariates included mental health factors such as stress and depression, as well as chronic conditions, including hypertension, diabetes, hyperlipidemia, stroke, renal failure, ischemic heart disease and cancer. BMI was calculated by dividing an individual's weight by the square of their height (kg/m^2^). Household income was categorized into quartiles: low (first quartile), lower‐middle (second quartile), upper‐middle (third quartile) and high (fourth quartile). Perceived stress was categorized as low or high. Hypertension was defined as systolic blood pressure ≥ 140 mmHg, diastolic blood pressure ≥ 90 mmHg or the use of antihypertensive medication. Diabetes mellitus was defined as fasting glucose > 126 mg/dL, the use of hypoglycemic medication or insulin, or a medical diagnosis of diabetes by a qualified physician. Participants who self‐reported a physician diagnosis of stroke, kidney failure, hyperlipidemia, depressive disorder, ischemic heart disease or cancer were classified as having the corresponding disease. A chronic condition count was calculated for each participant as the number of the following conditions reported (1, yes; 0, no): hypertension, dyslipidemia, diabetes mellitus, stroke, renal failure, ischemic heart disease and any type of cancer. This count was analysed both as a continuous and as a categorical variable with four levels: 0, 1, 2 and ≥ 3. The categorical approach was used to reflect the burden of multimorbidity while accounting for the low prevalence of individual conditions. The numbers and types of chronic conditions used to quantify chronic disease burden often vary across studies. This four‐level grouping system has been used in previous research to address multimorbidity [21]. Health behaviours, including smoking, alcohol consumption and physical activity, were assessed using the KNHANES questionnaire [14]. Participants were classified as current smokers (currently smoke or have smoked ≥ 100 cigarettes in their lifetime) or non‐current smokers (never smoked or smoked < 100 cigarettes in their lifetime). For alcohol consumption, participants were categorized as non‐drinkers (< 1 drink/week), moderate drinkers (1–13 drinks/week for men, 1–6 drinks/week for women) and heavy drinkers (≥ 14 drinks/week for men, ≥ 7 drinks/week for women). The number of drinks per week was calculated by multiplying the weekly frequency by the amount consumed per occasion. Physical activity metabolic equivalent task minutes (METs) were calculated by multiplying the weekly frequency of physical activity, the duration per day and an intensity constant for each activity (vigorous, 8; moderate, 4; light, 3.3). Vigorous activities included running, hiking, fast cycling, swimming, soccer, basketball, jumping rope, squash and carrying heavy objects. Moderate activities include slow swimming, doubles tennis, volleyball, badminton, table tennis and carrying light objects. Light activities included walking for at least 10 min. Physical activity levels were categorized based on total METs as low (< 600 METs/week), moderate (600–2999 METs/week) or vigorous (≥ 3000 METs/week) [22]. Covariates were selected a priori based on established vestibular epidemiology literature and their biological relevance to dizziness, vestibular dysfunction and musculoskeletal health [7, 8, 11, 23]. All selected variables were adjusted simultaneously in the multivariable models to minimize measured confounding.

### 2.4 Statistical Analysis

Statistical analyses were performed using SPSS for Windows, Version 26.0 (IBM Corp., Armonk, NY, USA). Descriptive statistics were used to summarize participant characteristics. Group comparisons were conducted using Rao–Scott chi‐squared tests for categorical variables and general linear models for continuous variables. Post hoc Bonferroni correction set the significance level for multinomial logistic regression at *p* < 0.017. Multinomial logistic regression was employed to evaluate the relationships between morning PV and various demographic, clinical and systemic factors. Models were adjusted for potential confounders, including age, sex, BMI, household income, chronic conditions, lifestyle factors and psychosocial variables. Odds ratios (ORs) and 95% confidence intervals (CIs) were calculated, and statistical significance was defined as a two‐tailed *p*‐value < 0.05. To confirm the robustness of the associations identified in the primary analysis, we conducted a sensitivity analysis. While the main analysis included participants who had experienced episodes of PV within the past year, the sensitivity analysis focused solely on those with active, ongoing PV at the time of the survey. As in the primary analysis, vestibular impairment was defined as failure of condition 4 on the modified Romberg test, and participants were grouped according to whether they passed or failed this condition. All statistical analyses and covariate adjustments were consistent with those used in the primary analysis. However, all participants in the current PV ^*abn*^ *Romberg* group had abnormal BMD. Therefore, categorical analysis was precluded due to complete separation, and BMD was analysed using the min T‐score as a continuous variable. Analyses were weighted and adjusted to reflect the complex sampling design. To examine potential age‐related effect modification, we additionally performed age‐stratified multinomial logistic regression using 65 years as the cutoff. Because the number of vestibular‐impaired current PV cases was small (*n* = 29), age stratification was not feasible in this subset, and age was modelled only as a continuous covariate. To estimate values representative of the general population outside institutions, all analyses used survey sample weights calculated based on the sampling rate, response rate and age/sex proportions of the reference population (2005 Korean Population Census). Following KNHANES analytic guidelines, user‐missing codes (e.g., non‐response, unknown) were retained to preserve sampling weights and complex‐survey design variables. However, observations with missing values in analytic covariates required for regression modelling were automatically excluded by the complex‐sample procedures in SPSS (i.e., default complete‐case analysis). These exclusions are reflected in Figure 1 [24].

### 2.5 Ethics Statement

The KNHANES protocols were reviewed and approved by the Institutional Review Board (IRB) of the Korea Disease Control and Prevention Agency (IRB Nos. 2008‐04EXP‐01‐C, 2009‐01CON‐032C, 2010‐02CON‐21‐C), and the study was conducted in accordance with the tenets of the 1964 Declaration of Helsinki and its later amendments. Written informed consent was obtained from all participants for each KNHANES cycle. This study was deemed exempt from additional IRB review by the Chungnam National University Hospital IRB (IRB No. 2025‐08‐042) under Article 13 of the Enforcement Rules of the Bioethics and Safety Act because it analysed existing records without collecting or recording any personally identifiable information.

## 3 Results

### 3.1 Prevalence of Morning PV

**FIGURE 2:**
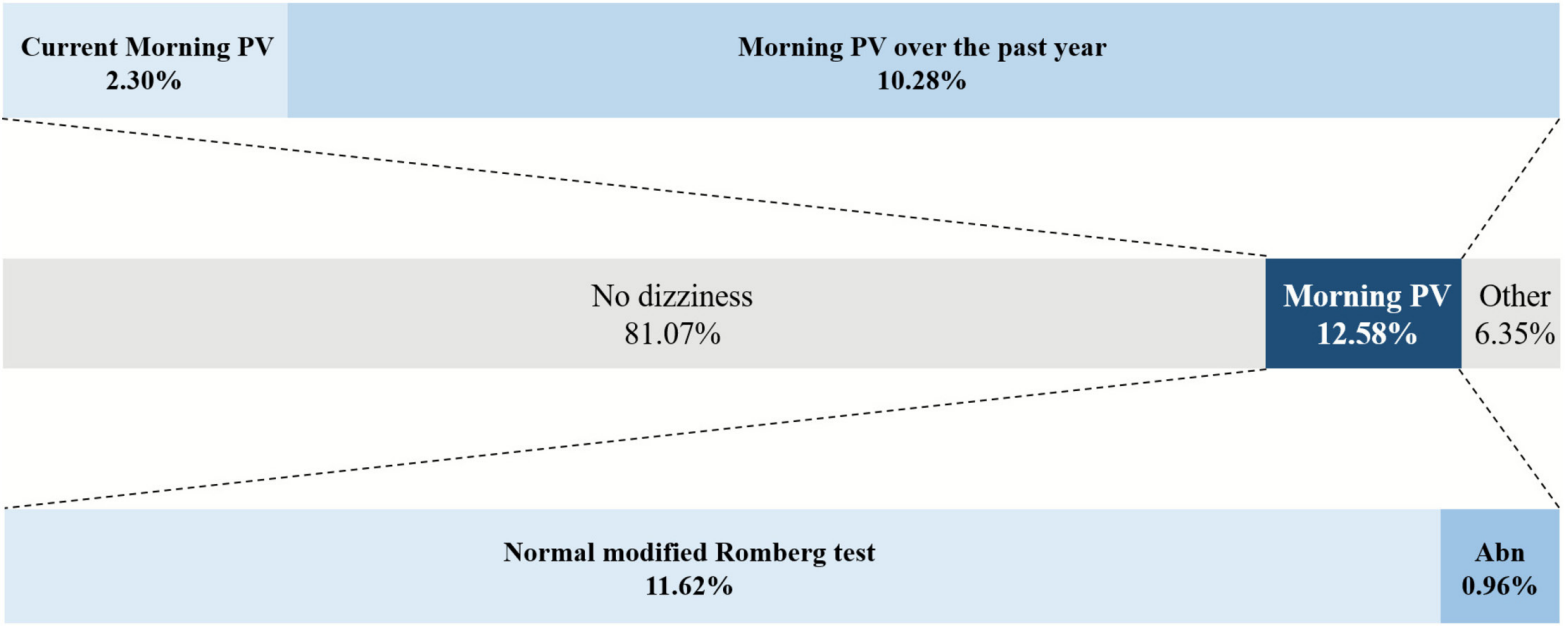
Flow diagram illustrating the prevalence and stratification of morning PV among Korean adults aged ≥ 40 years. Participants were initially categorized into three groups based on self‐reported dizziness symptoms: no dizziness (81.07%), morning PV (12.58%) and other types of dizziness (6.35%). Among those with morning PV, 10.28% reported symptoms during the past year, and 2.30% reported current symptoms at the time of the survey. Vestibular function was assessed using the modified Romberg test, and these results were used to further stratify the morning PV group into individuals with normal (11.62%) and abnormal (0.96%) vestibular function. This stratification enabled the identification of participants with morning PV attributable to vestibular impairment, potentially reflecting subclinical or persistent vestibular dysfunction. abn, abnormal; PV, positional vertigo.

Among the 8512 participants included in the final analysis, the overall 1‐year prevalence of dizziness was 18.93% (95% CI, 17.58–20.35) (Figure 2, Online Resource 1). Morning PV, defined as severe dizziness upon turning in bed or rising in the morning, was reported by 12.58% (95% CI, 11.38–13.89) of participants, making it the most common dizziness subtype. Abnormal Romberg performance (^*abn*^ *Romberg*), indicating vestibular dysfunction, was present in 0.96% of all survey respondents and in 7.66% of those with morning PV. Current symptoms were reported by 2.30% (95% CI, 1.91–2.77) of participants, reflecting an active clinical state of PV, whereas 10.28% (95% CI, 9.26–11.40) reported only previous episodes over the past year. Other types of dizziness that did not meet the criteria for morning PV were reported by 6.35% (*n* = 583; 95% CI, 5.50–7.31). The remaining 81.07%, who reported no dizziness or imbalance within the past year and passed the modified Romberg test, constituted the control group with intact vestibulo‐somatosensory function.

### 3.2 Body Composition Differences by Functional Balance Status

**FIGURE 3:**
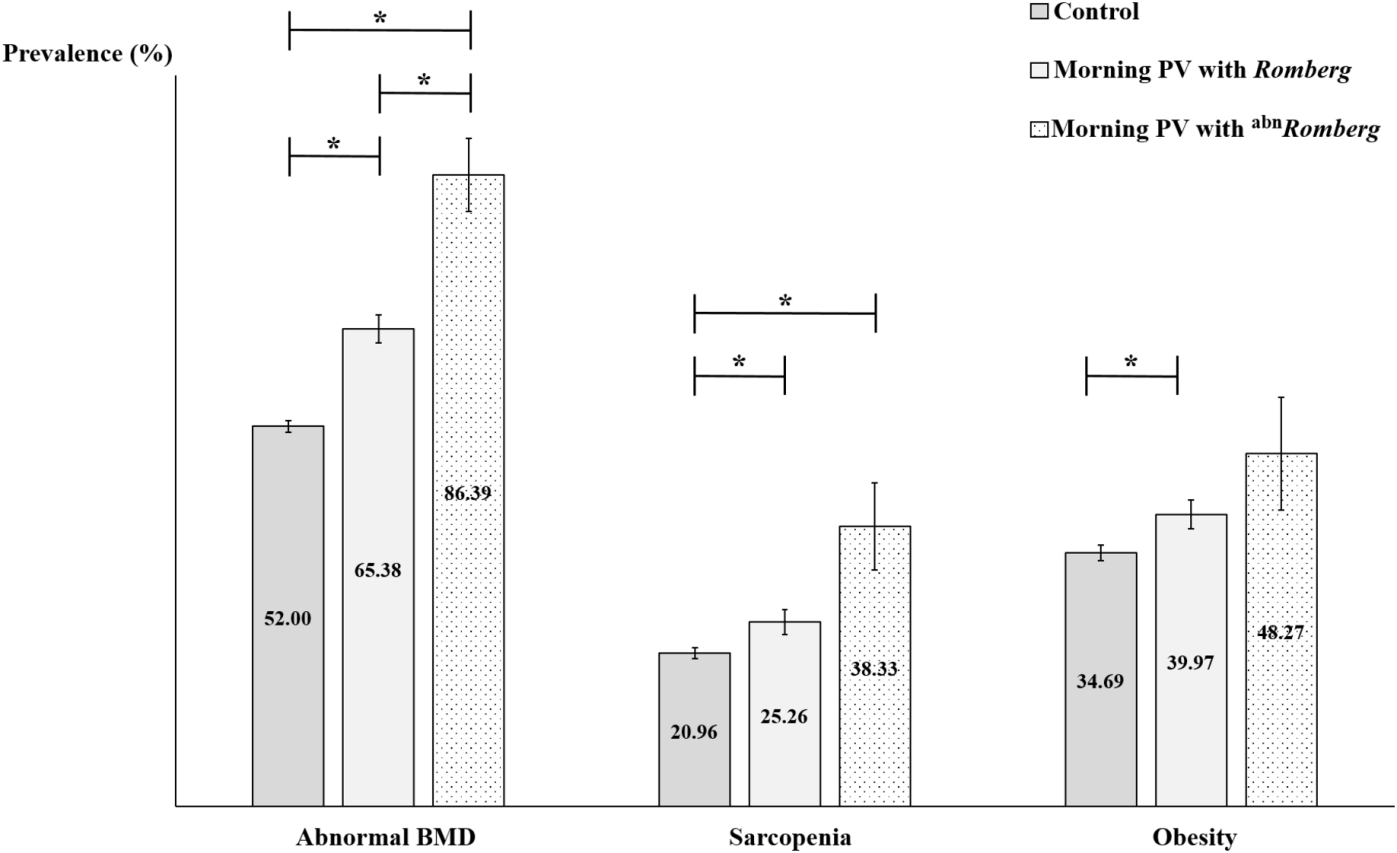
Comparison of the prevalence of osteopenia/osteoporosis, sarcopenia and obesity according to vestibular function status among participants with morning PV and controls. Participants were classified based on their dizziness symptoms and performance on Condition 4 of the modified Romberg test (standing on a compliant foam surface with eyes closed) into three groups: a control group (no dizziness), morning PV with normal Romberg test results (morning PV and *Romberg*) and morning PV with abnormal Romberg test results (morning PV and ^*abn*^ *Romberg*). The prevalence of abnormal BMD was highest in the morning PV and ^*abn*^ *Romberg* group (86.39%), followed by the morning PV and *Romberg* group (65.38%) and lowest in the control group (52.00%). Sarcopenia prevalence was higher in the morning PV and ^*abn*^ *Romberg* (38.33%) and morning PV and *Romberg* (25.26%) groups compared with controls (20.96%). Obesity was significantly more prevalent in the morning PV and *Romberg* group (39.97%) than in controls (34.69%). Bars represent weighted prevalence, and error bars indicate standard errors. Asterisks (*) denote statistically significant differences between groups based on post hoc comparisons (*p* < 0.017). abn, abnormal; BMD, bone mineral density; PV, positional vertigo.

The weighted prevalence of abnormal BMD, sarcopenia and obesity differed across the three groups (Figure 3). Abnormal BMD was most prevalent in the ^*abn*^ *Romberg* group (86.39%), followed by the *Romberg* group (65.38%) and the control group (52.00%). Sarcopenia was more common in the ^*abn*^ *Romberg* (38.33%) and *Romberg* (25.26%) groups than in the control group (20.96%). The prevalence of obesity was higher in the *Romberg* group (39.97%) than in the control group (34.69%). Analyses of BMD, muscle mass and fat mass as continuous variables demonstrated trends consistent with these categorical prevalence estimates (Online Resource 2).

### 3.3 Sociodemographic and Behavioural Profiles

The three groups, stratified by vestibular function, showed distinct sociodemographic, psychosocial and lifestyle characteristics (Online Resource 3).

Participants in the ^abn^ *Romberg* group tended to be older and were more likely to come from lower‐income households. The proportion of women was higher in this group than in the other two groups (Online Resource 3). Depression and high perceived stress were most common in the ^a*bn*^ *Romberg* group, followed by the *Romberg* group and then the control group (all *p* < 0.001). Low physical activity was also most prevalent in the ^*abn*^ *Romberg* group (39.34%), and non‐drinking was particularly frequent in this group (77.83%). Smoking prevalence was lower in both PV groups than in the control group (all *p* < 0.05).

### 3.4 Comorbidity by Functional Balance Status

The burden of chronic conditions, defined as the number of concurrent diseases, varied between groups (Online Resource 2). The ^*abn*^ *Romberg* group had the highest comorbidity rate (1.46 ± 0.12), with nearly half (47.67%) having at least two chronic conditions and only 13.40% having none (*p* < 0.001) (Online Resource 3).

### 3.5 Multivariable Associations Between Systemic Factors and Morning PV

**FIGURE 4:**
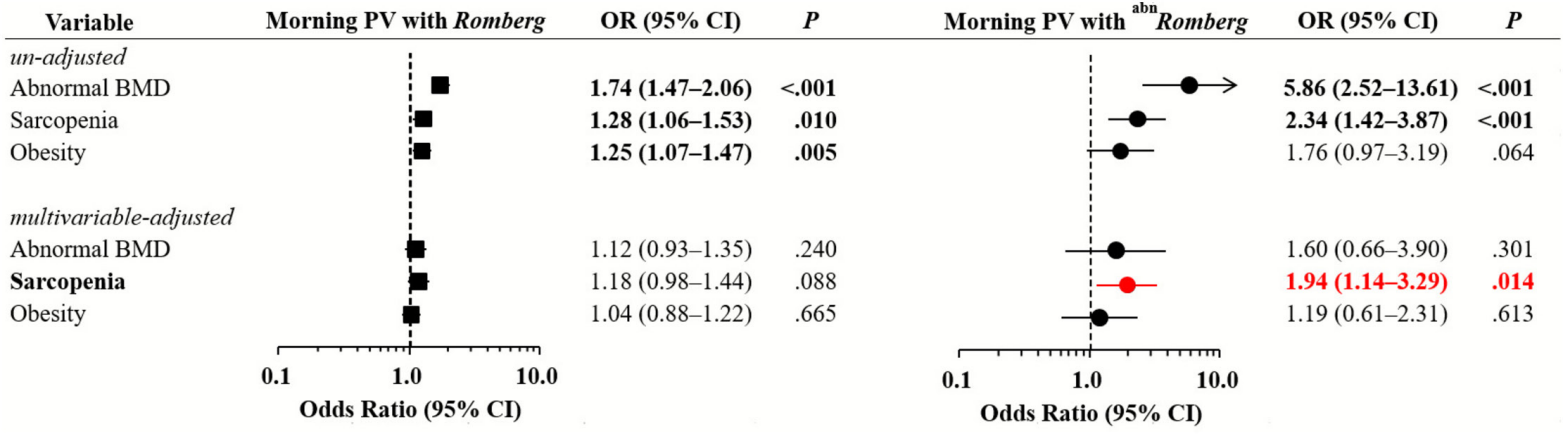
Multinomial logistic regression analysis of the relationships between body composition variables and morning PV, stratified by Romberg test status. Forest plots showing the ORs and 95% CIs from multinomial logistic regression assessing associations between body composition variables (abnormal BMD, sarcopenia and obesity) and morning PV, stratified by Romberg test results. Black squares and circles represent the ORs for the morning PV and *Romberg* and morning PV and ^abn^ *Romberg* groups, respectively. Horizontal lines through each marker indicate 95% CIs. Unadjusted ORs are shown above, and multivariable‐adjusted ORs are shown below (adjusted for sex, age, household income, depressive disorder, physical activity, alcohol consumption, smoking status, perceived stress and number of chronic conditions). The control group was used as the reference category. Red markers indicate statistically significant results (*p* < 0.05) in the multivariable analysis. Arrows indicate CIs extending beyond the plotted range. abn, abnormal; BMD, bone mineral density; CI, confidence interval; OR, odds ratio; PV, positional vertigo.

Multinomial multivariable logistic regression, with the control group serving as the reference, revealed distinct risk profiles for morning PV with and without vestibular dysfunction (Figure 4, Online Resource 4). In the ^*abn*^ *Romberg* group, sarcopenia was independently associated with increased odds of morning PV (OR = 1.94; 95% CI, 1.14–3.29; *p* = 0.014), whereas this association was not significant in the *Romberg* group (OR = 1.18; 95% CI, 0.98–1.44; *p* = 0.088). This suggests that muscle mass depletion may play a particularly important role in morning PV among individuals with impaired postural control (Figure 4). Other significant predictors in the *abnRomberg* group included older age (OR = 1.09; 95% CI, 1.06–1.12), female sex (OR = 2.28; 95% CI, 1.18–4.41), depression (OR = 2.74; 95% CI, 1.46–5.15) and high perceived stress (OR = 1.75; 95% CI, 1.11–2.75). After adjustment, abnormal BMD and obesity were not significantly associated with morning PV in this subgroup. The *Romberg* group showed a different pattern. Significant predictors included older age (OR = 1.02; 95% CI, 1.01–1.03), female sex (OR = 1.97; 95% CI, 1.61–2.41), lower household income (Q1 vs. Q4: OR = 1.52; 95% CI, 1.16–1.99), depression (OR = 1.54; 95% CI, 1.14–2.09), high perceived stress (OR = 1.71; 95% CI, 1.44–2.04) and current smoking (OR = 1.29; 95% CI, 1.01–1.64). Notably, heavy drinking was inversely associated with morning PV in this group (OR = 0.72; 95% CI, 0.54–0.96).

### 3.6 Sensitivity Analysis

**FIGURE 5:**
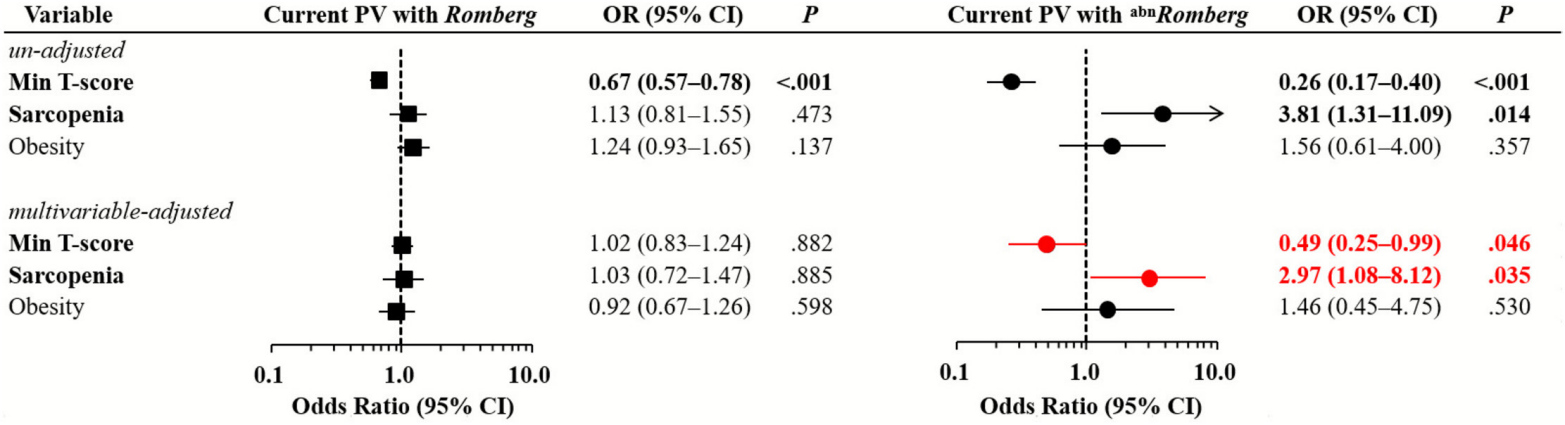
Sensitivity analysis of body composition variables and current PV according to Romberg test status. Forest plots showing the ORs and 95% CIs from multinomial logistic regression assessing associations between body composition variables (min T‐score, sarcopenia and obesity) and current PV, stratified by Romberg test results. Only participants reporting current PV (2.30%) were included. The min T‐score was used instead of abnormal BMD, as all current PV cases with ^*abn*^ *Romberg* were associated with abnormal BMD. Black squares and circles represent the ORs for current PV and *Romberg* and current PV and ^abn^ *Romberg*, respectively. Horizontal lines through each marker indicate 95% CIs. Unadjusted ORs are shown above, and multivariable‐adjusted ORs are shown below (adjusted for age, sex, household income, depressive disorder, physical activity, alcohol consumption, smoking status, perceived stress and number of chronic conditions). The control group was used as the reference category. Red markers indicate statistically significant results (*p* < 0.05) in the multivariable analysis. Arrows indicate CIs extending beyond the plotted range. abn, abnormal; BMD, bone mineral density; CI, confidence interval; OR, odds ratio; PV, positional vertigo.

A sensitivity analysis was conducted including only participants with current PV, stratified by Romberg status and compared with the control group. As in the primary analysis, sarcopenia remained independently associated with vestibular impairment in the ^*abn*^ *Romberg* group of this subset (OR = 2.97; 95% CI, 1.08–8.12; *p* = 0.035) (Figure 5). Categorical BMD status was not included due to complete data separation, as all participants in the ^*abn*^ *Romberg* group had osteopenia or osteoporosis. Instead, the min T‐score was used as a continuous variable and was independently associated with ^*abn*^ *Romberg* (OR = 0.49; 95% CI, 0.25–0.99; *p* = 0.046). This suggests that compromised bone integrity may contribute to vestibular dysfunction in the presence of active symptoms (Figure 5, Online Resource 5). Older age (OR = 1.09 per year; 95% CI, 1.03–1.16; *p* = 0.006) and low physical activity (< 600 METs/week) (OR = 0.28; 95% CI, 0.09–0.92; *p* = 0.036) were also independently associated with vestibular‐impaired current PV. Female sex, a diagnosis of depression, high perceived stress and a higher comorbidity burden were not significantly associated with vestibular impairment in this subgroup. In an additional age‐stratified analysis (cutoff 65 years), sarcopenia was associated with vestibular‐impaired morning PV only among adults aged ≥ 65 years, whereas T‐scores were not significant (Online Resources 6 and 7). Age stratification was not feasible for the current PV due to the small number of vestibular‐impaired cases.

## 4 Discussion

In this nationally representative study of Korean adults aged ≥ 40 years, we identified novel associations between morning PV, vestibular impairment and systemic factors such as sarcopenia and bone status. These findings extend the literature on BPPV, for which prevalence estimates have traditionally ranged from 1.60% to 2.40% [25]. The substantially higher clinical burden of morning PV in our population suggests that atypical or central variants of PV, as well as under‐recognition in clinical practice, may contribute to a larger reservoir of unaddressed positional dizziness in the community [26, 27, 28]. Among individuals with morning PV, 7.1% demonstrated vestibular impairment, and 20.3% reported active symptoms, underscoring its clinical relevance.

A key finding of this study is the specific association between vestibular dysfunction and sarcopenia in individuals with morning PV. Sarcopenia remained independently associated with vestibular‐impaired morning PV across multivariable models and sensitivity analyses, with stronger associations observed in older adults, suggesting that aging may amplify the interplay between muscle decline and vestibular dysfunction. Several biological pathways support this observation. Vestibular dysfunction may influence muscle metabolism through alterations in hypothalamic melanocortin signalling and autonomic regulation [29, 30, 31]. The vestibulo‐autonomic network contributes to muscle perfusion and antigravity tone, and impaired vestibular input to autonomic centres—including the dorsal motor nucleus of the vagus (DMV) and nucleus of the solitary tract (NTS)—may affect systemic inflammatory and metabolic responses [31, 32, 33]. Additionally, vestibular inputs facilitate muscle activation and postural control [34, 35, 36], whereas sarcopenia‐related neuromuscular decline may hinder vestibular compensation [37, 38]. Taken together, these findings support a bidirectional framework in which vestibular impairment and sarcopenia may mutually reinforce one another. The clustering of sarcopenia and low BMD among individuals with abnormal Romberg performance further suggests contributions from both direct vestibulo‐autonomic mechanisms and indirect, balance‐mediated pathways. Lower BMD was also associated with vestibular‐impaired morning PV, consistent with previous reports linking osteopenia and osteoporosis to BPPV [10]. These findings underscore the importance of considering bone health when evaluating persistent or recurrent PV and suggest that compromised bone integrity may increase susceptibility to instability or impair compensatory mechanisms [10, 39]. Further prospective research is warranted to explore the clinical significance of these associations and their potential implications for managing recurrent or persistent PV. Although the vestibular‐impaired PV subgroup was relatively small (*n* = 91), the reproducibility of associations for both sarcopenia and low BMD in sensitivity analyses restricted to active PV strengthens the credibility of these observations; nevertheless, subgroup findings should be interpreted cautiously.

Despite the robust design, use of nationally representative data and comprehensive body composition analysis, this study has several limitations. Its cross‐sectional design precludes causal inference, and the use of self‐reported dizziness introduces potential recall bias. Temporal relationships cannot be determined, and the observed associations should be interpreted as correlational. Longitudinal or mechanistic studies are needed to clarify whether musculoskeletal deficits predispose individuals to vestibular‐impaired PV or whether recurrent vertigo contributes to systemic frailty. Moreover, the absence of definitive diagnostic tools for BPPV, such as the Dix–Hallpike test, limited a more precise characterization of PV. In addition, vestibular dysfunction was inferred using the modified Romberg test rather than being directly confirmed by vestibular evaluations such as caloric testing, vestibular evoked myogenic potential (VEMP) or video head impulse testing (vHIT). Romberg‐based postural tests have shown moderate diagnostic accuracy for vestibular disorders, with sensitivity and specificity of roughly 70%–80%, and the foam–eyes‐closed condition used in the modified Romberg test demonstrates comparable performance in prior studies. However, no studies have directly compared the modified Romberg test with vHIT or VEMP [40].

This may limit the diagnostic specificity of our classifications. However, the modified Romberg test has been widely used as a functional surrogate in previous large‐scale epidemiologic studies and constitutes a feasible strategy for community‐based vestibular screening when formal testing is not routinely available [7, 8, 16, 17]. Additionally, some potentially relevant confounders—such as medication effects, micronutrient deficiencies and more granular physical activity metrics—were not fully captured. Missing data and complete‐case analysis may also introduce selection bias, although the use of survey weights and evidence from previous KNHANES vestibular studies support the generalizability of our findings. Given the high prevalence and systemic correlates of morning PV, incorporating brief screening for sarcopenia, bone health and physical activity into routine care may help identify individuals at heightened risk for vestibular impairment. Morning PV accompanied by impaired postural stability may serve as an early indicator of broader systemic vulnerability, particularly in older adults. Targeted strategies—including vestibular rehabilitation, resistance training and nutritional optimization—may therefore offer dual benefits for musculoskeletal health and vestibular function. Future prospective, mechanistic and interventional studies are warranted to determine whether modifying these factors can alter the trajectory of recurrent or persistent morning PV.

## Funding

This work was supported by the research fund of Chungnam National University (2025‐1148‐01).

## Conflicts of Interest

The authors declare no conflicts of interest.

## Supporting information


**Table S1:** Stratified prevalence of morning positional vertigo by symptom status and balance test performance.
**Table S2:** Clinical characteristics of morning positional vertigo stratified by modified Romberg test results.
**Table S3:** Demographic and health‐related characteristics of morning positional vertigo stratified by modified Romberg test results.
**Table S4:** Multinomial logistic regression analysis of morning positional vertigo according to modified Romberg test status.
**Table S5:** Sensitivity analysis of multinomial logistic regression for current positional vertigo.
**Table S6:** Age‐stratified sensitivity analysis of multinomial logistic regression by age group (< 65 years).
**Table S7:** Age‐stratified sensitivity analysis of multinomial logistic regression by age group (≥ 65 years).

## Data Availability

The data used in this study are publicly available from the Korea Disease Control and Prevention Agency via the KNHANES website. Website registration is required to access the dataset in SAS/SPSS format (https://knhanes.kdca.go.kr/knhanes/eng/main.do). All data have been de‐identified and are freely accessible for research purposes.
