# Supplementary material for: Association of Sarcopenia and Lower Bone Density With Positional Vertigo in the Morning: Insights From a Nationwide Survey
Source: J Cachexia Sarcopenia Muscle. 2026 Feb 3;17(1):e70219. doi: 10.1002/jcsm.70219 (PMC12867951; doi:10.1002/jcsm.70219)
Supplement: Supplementary file 1 — Table S1: Stratified prevalence of morning positional vertigo by symptom status and balance test performance. Table S2: Clinical characteristics of morning positional vertigo stratified by modified Romberg test results. Table S3: Demographic and health‐related characteristics of morning positional vertigo stratified by modified Romberg test results. Table S4: Multinomial logistic regression analysis of morning positional vertigo according to modified Romberg test status. Table S5: Sensitivity analysis of multinomial logistic regression for current positional vertigo. Table S6: Age‐stratified sensitivity analysis of multinomial logistic regression by age group (< 65 years). Table S7: Age‐stratified sensitivity analysis of multinomial logistic regression by age group (≥ 65 years). [file JCSM-17-e70219-s001.docx]

**Supplementary Table 1. Stratified Prevalence of Morning Positional Vertigo by Symptom Status and Balance Test Performance**

|  | **Number** | **Weighted %** | **95% CI** | **SE** |
| --- | --- | --- | --- | --- |
| **Dizziness** | 1,866 | 18.93 | 17.58–20.35 | 0.70 |
| **Morning PV** | 1,283 | 12.58 | 11.38–13.89 | 0.64 |
| Current PV | 261 | 2.30 | 1.91–2.77 | 0.22 |
| current PV with *Romberg* | 232 | 1.99 | 1.64–2.41 | 0.20 |
| current PV with *^abn^Romberg* | 29 | 0.31 | 0.18–0.52 | 0.08 |
| Previous PV | 1,022 | 10.28 | 9.26–11.40 | 0.54 |
| previous PV with *Romberg* | 960 | 9.62 | 8.70–10.64 | 0.49 |
| previous PV with *^abn^Romberg* | 62 | 0.65 | 0.44–0.98 | 0.13 |
| Summary (*Romberg* results) |  |  |  |  |
| Morning PV with *Romberg* | 1,192 | 11.62 | 10.55–12.77 | 0.56 |
| Morning PV with *^abn^Romberg* | 91 | 0.96 | 0.66–1.40 | 0.19 |
| **Other dizziness** | 583 | 6.35 | 5.50–7.31 | 0.46 |

Abbreviations: CI = Confidence interval; SE = Standard error**;** PV = Positional vertigo; abn = Abnormal.

Morning positional vertigo (PV) was defined as severe vertigo when turning in bed or rising in the morning within the past year.

Current PV indicated ongoing symptoms; previous PV indicated past-year symptoms only.

Other dizziness referred to participants with dizziness or balance problems who did not meet criteria for morning PV.

^abn^Romberg denoted failure on condition 4 (standing on compliant foam with eyes closed), indicating vestibular dysfunction.

**Supplementary Table 2. Clinical Characteristics of Morning Positional Vertigo Stratified by Modified Romberg Test Results**

|  | **^a^Control (n=7,229)** | **^b^Morning PV with *Romberg* (n=1,192)** | **^c^Morning PV with *^abn^Romberg* (n=91)** | ***p* value** | *Post hoc comparisons |
| --- | --- | --- | --- | --- | --- |
| **Age** | **53.45±0.19** | **57.32±0.46** | **66.47±1.49** | **<0.001** | a<b<c |
| **BMI (kg/m^2^)** | **24.10±0.04** | **23.79±0.12** | **23.33±0.58** | **0.031** | b<a |
| **Body composition** |  |  |  |  |  |
| ASM (kg) | **18.40±0.06** | **16.42±0.18** | **14.90±0.42** | **<0.001** | c<b<a |
| BFM (kg) | 17.45±0.10 | 17.95±0.20 | 17.60±1.06 | 0.150 |  |
| ASMI (kg/m^2^) | **6.88±0.02** | **6.42±0.05** | **6.08±0.11** | **<0.001** | c<b<a |
| BFM percentage (%) | **27.67±0.14** | **30.08±0.32** | **30.69±1.35** | **<0.001** | a<b |
| Total femur T-score | **0.08±0.02** | **-0.20±0.04** | **-1.08±0.17** | **<0.001** | c<b<a |
| Femoral neck T-score | **-0.72±0.02** | **-1.09±0.04** | **-1.94±0.17** | **<0.001** | c<b<a |
| Lumbar spine T-score | **-0.72±0.02** | **-1.09±0.05** | **-1.86±0.16** | **<0.001** | c<b<a |
| ^d^Min T-score | **-1.08±0.02** | **-1.45±0.04** | **-2.28±0.16** | **<0.001** | c<b<a |
| **^e^Chronic condition count** | **1.09±0.02** | **1.20±0.04** | **1.46±0.12** | **<0.001** | a<b,c |

Abbreviations: PV = Positional vertigo; abn = Abnormal; BMI = Body mass index; ASM = Appendicular skeletal muscle mass; BFM = Body fat mass; ASMI = Appendicular skeletal muscle mass index.

Superscripts a–c were used to indicate group labels for post hoc comparisons, while d–e referred to variable definitions.

^d^Minimum T-score referred to the lowest value among the total femur, femoral neck, and lumbar spine T-scores.
^e^Chronic condition count indicated the total number of physician-diagnosed chronic diseases, including hypertension, dyslipidemia, diabetes mellitus, stroke, chronic kidney disease, ischemic heart disease (myocardial infarction or angina), and any type of cancer.

^abn^Romberg denoted failure on condition 4 (standing on compliant foam with eyes closed), indicating vestibular dysfunction.
*Multiple comparisons were conducted using Bonferroni correction (p value <0.017).

Values were presented as weighted means ± standard error.

Bold values indicated statistical significance (*p* value <0.05).

**Supplementary Table 3. Demographic and Health-Related Characteristics of Morning Positional Vertigo Stratified by Modified Romberg Test Results**

| **Variable** | **Control**  **(n=7,229)** | | **Morning PV with *Romberg* (n=1,192)** | | **Morning PV with *^abn^Romberg* (n=91)** | | ***p* value** |
| --- | --- | --- | --- | --- | --- | --- | --- |
|  | **Number** | **Weight %(SE)** | **Number** | **Weight %(SE)** | **Number** | **Weight %(SE)** |  |
| **Sex** |  |  |  |  |  |  | **<0.001** |
| men | 3,416 | 52.70 (0.58) | 339 | 32.99 (1.76) | 26 | 25.10 (5.09) |  |
| women | 3,813 | 47.30 (0.58) | 853 | 67.01 (1.76) | 65 | 74.90 (5.09) |  |
| **Household income** |  |  |  |  |  |  | **<0.001** |
| Q1 (low) | 1,559 | 17.68 (0.74) | 419 | 30.57 (1.88) | 50 | 45.36 (5.39) |  |
| Q2 (lower-middle) | 1,761 | 24.57 (0.82) | 285 | 24.36 (1.77) | 22 | 21.80 (5.42) |  |
| Q3 (upper-middle) | 1,887 | 27.93 (0.79) | 255 | 23.13 (1.54) | 10 | 17.12 (5.27) |  |
| Q4 (higher) | 2,022 | 29.83 (1.04) | 233 | 21.94 (1.71) | 9 | 15.72 (5.54) |  |
| **Hypertension** |  |  |  |  |  |  | **0.001** |
| normal | 2,982 | 43.87 (0.88) | 447 | 40.23 (1.98) | 18 | 20.39 (5.57) |  |
| prehypertension, hypertension | 4,247 | 56.13 (0.88) | 745 | 59.77 (1.98) | 73 | 79.61 (5.57) |  |
| Diabetes mellitus |  |  |  |  |  |  | 0.094 |
| normal | 4,625 | 64.11 (0.72) | 778 | 65.43 (1.65) | 50 | 52.35 (5.95) |  |
| pre-diabetes, diabetes | 2,604 | 35.89 (0.72) | 414 | 34.57 (1.65) | 41 | 47.65 (5.95) |  |
| **Diagnosis of stroke** |  |  |  |  |  |  | **0.004** |
| no | 7,107 | 98.76 (0.15) | 1,150 | 97.54 (0.44) | 89 | 98.16 (1.34) |  |
| yes | 122 | 1.24 (0.15) | 42 | 2.46 (0.44) | 2 | 1.84 (1.34) |  |
| Diagnosis of kidney failure |  |  |  |  |  |  | 0.053 |
| no | 7,217 | 99.86 (0.05) | 1,185 | 99.59 (0.18) | 90 | 99.41 (0.60) |  |
| yes | 12 | 0.14 (0.05) | 7 | 0.41 (0.18) | 1 | 0.59 (0.60) |  |
| **Diagnosis of depression disorder** |  |  |  |  |  |  | **<0.001** |
| no | 6,974 | 97.00 (0.22) | 1,107 | 93.53 (0.80) | 78 | 88.07 (3.12) |  |
| yes | 255 | 3.00 (0.22) | 85 | 6.47 (0.80) | 13 | 11.93 (3.12) |  |
| **Diagnosis of hyperlipidemia** |  |  |  |  |  |  | **0.008** |
| no | 6,419 | 89.70 (0.42) | 1,009 | 86.12 (1.15) | 80 | 90.90 (3.68) |  |
| yes | 810 | 10.30 (0.42) | 183 | 13.88 (1.15) | 11 | 9.10 (3.68) |  |
| **Physical activity** |  |  |  |  |  |  | **0.012** |
| low (<600 ^a^METs/week) | 1,963 | 26.85 (0.78) | 383 | 30.15 (1.74) | 41 | 39.34 (7.69) |  |
| moderate (600 – 2,999 METs/week) | 2,928 | 40.16 (0.76) | 488 | 42.54 (1.81) | 30 | 33.15 (6.08) |  |
| vigorous (≥3,000 METs/week) | 2,338 | 32.99 (0.84) | 321 | 27.31 (1.67) | 20 | 27.51 (5.65) |  |
| **Alcohol drinking** |  |  |  |  |  |  | **<0.001** |
| none (<1 drink/week) | 3,740 | 46.81 (0.71) | 797 | 62.66 (1.84) | 68 | 77.83 (5.12) |  |
| moderate (1–13 for men, 1–6 for women) | 2,103 | 30.79 (0.68) | 264 | 24.11 (1.60) | 17 | 16.51 (4.89) |  |
| heavy (≥14 for men, ≥7 for women) | 1,386 | 22.40 (0.60) | 131 | 13.23 (1.41) | 6 | 5.65 (2.25) |  |
| **Current smoking status** |  |  |  |  |  |  | **0.002** |
| no (never smoker, ex-smoker) | 5,781 | 76.02 (0.64) | 1,021 | 81.30 (1.62) | 75 | 86.27 (4.06) |  |
| current smoker | 1,448 | 23.98 (0.64) | 171 | 18.70 (1.62) | 16 | 13.73 (4.06) |  |
| **Perceived stress** |  |  |  |  |  |  | **<0.001** |
| low (rare/mild) | 5,526 | 75.57 (0.60) | 767 | 63.37 (1.75) | 55 | 63.04 (5.24) |  |
| high (moderate/severe) | 1,703 | 24.43 (0.60) | 425 | 36.63 (1.75) | 36 | 36.96 (5.24) |  |
| **Diagnosis of ischemic heart disease** |  |  |  |  |  |  | **<0.001** |
| no | 7,045 | 98.14 (0.18) | 1,135 | 95.53 (0.70) | 90 | 99.65 (0.35) |  |
| yes | 184 | 1.86 (0.18) | 57 | 4.47 (0.70) | 1 | 0.35 (0.35) |  |
| Diagnosis of cancer |  |  |  |  |  |  | 0.108 |
| no | 6,965 | 96.91 (0.24) | 1,140 | 95.83 (0.72) | 85 | 93.07 (3.63) |  |
| yes | 264 | 3.09 (0.24) | 52 | 4.17 (0.72) | 6 | 6.93 (3.63) |  |
| **^b^Chronic condition count** |  |  |  |  |  |  | **<0.001** |
| 0 | 2,031 | 30.28 (0.76) | 292 | 27.33 (1.69) | 11 | 13.40 (5.12) |  |
| 1 | 2,748 | 37.68 (0.74) | 446 | 36.33 (1.72) | 37 | 38.93 (5.41) |  |
| 2 | 1,931 | 25.98 (0.67) | 324 | 26.62 (1.45) | 33 | 36.93 (5.38) |  |
| ≥3 | 519 | 6.06 (0.34) | 130 | 9.72 (1.16) | 10 | 10.74 (4.54) |  |

Abbreviations: PV = Positional vertigo; abn = Abnormal; SE = Standard error; METs = Metabolic Equivalent Task-minutes.

^a^MET: multiplying the days of physical activity per week by the duration (minutes) per day by a constant determined for each activity (vigorous activity: 8, moderate activity: 4, and walking: 3.3)

**^b^Chronic condition count** was categorized as 0, 1, 2, or ≥3 based on the total number of physician-diagnosed chronic diseases (e.g., hypertension, diabetes, cancer).

^abn^Romberg denoted failure on condition 4 (standing on compliant foam with eyes closed), indicating vestibular dysfunction.

Values were presented as unweighted numbers and weighted percentages (standard error), based on the complex sampling design.

Bold values indicated significance (*p* value <0.05).

**Supplementary Table 4. Multinomial Logistic Regression Analysis of Morning Positional Vertigo According to Modified Romberg Test Status**

| **Variable** | **Morning PV with *Romberg*** | | | **Morning PV with *^abn^Romberg*** | | |
| --- | --- | --- | --- | --- | --- | --- |
|  | **OR** | **95% CI** | ***p* value** | **OR** | **95% CI** | ***p* value** |
| **Age** | **1.02** | **1.01–1.03** | **<0.001** | **1.09** | **1.06–1.12** | **<0.001** |
| **Sex** |  |  |  |  |  |  |
| men | 1.00 |  |  | 1.00 |  |  |
| **women** | **1.97** | **1.61–2.41** | **<0.001** | **2.28** | **1.18–4.41** | **0.014** |
| **Household income** |  |  |  |  |  |  |
| **Q1 (low)** | **1.52** | **1.16–1.99** | **0.003** | 1.32 | 0.62–2.81 | 0.463 |
| Q2 (lower-middle) | 1.18 | 0.91–1.52 | 0.203 | 1.05 | 0.41–2.73 | 0.917 |
| Q3 (upper-middle) | 1.11 | 0.88–1.40 | 0.396 | 1.04 | 0.38–2.87 | 0.933 |
| Q4 (higher) | 1.00 |  |  | 1.00 |  |  |
| ^a^Abnormal BMD |  |  |  |  |  |  |
| normal | 1.00 |  |  | 1.00 |  |  |
| abnormal BMD | 1.12 | 0.93–1.35 | 0.240 | 1.60 | 0.66–3.90 | 0.301 |
| **^b^Sarcopenia** |  |  |  |  |  |  |
| normal | 1.00 |  |  |  |  |  |
| **sarcopenia** | 1.18 | 0.98–1.44 | 0.088 | **1.94** | **1.14–3.29** | **0.014** |
| ^c^Obesity |  |  |  |  |  |  |
| normal | 1.00 |  |  | 1.00 |  |  |
| obesity | 1.04 | 0.88–1.22 | 0.665 | 1.19 | 0.61–2.31 | 0.613 |
| **Diagnosis of depression disorder** |  |  |  |  |  |  |
| no | 1.00 |  |  |  |  |  |
| **yes** | **1.54** | **1.14–2.09** | **0.005** | **2.74** | **1.46–5.15** | **0.002** |
| Physical activity |  |  |  |  |  |  |
| low (<600 ^d^METs/week) | 1.09 | 0.89–1.35 | 0.403 | 1.02 | 0.46–2.30 | 0.955 |
| moderate (600 – 2,999 METs/week) | 1.15 | 0.94–1.40 | 0.176 | 0.72 | 0.38–1.36 | 0.313 |
| vigorous (≥3,000 METs/week) | 1.00 |  |  | 1.00 |  |  |
| **Alcohol drinking** |  |  |  |  |  |  |
| none (<1 drink/week) | 1.00 |  |  | 1.00 |  |  |
| moderate (1–13 for men, 1–6 for women) | 0.83 | 0.67–1.01 | 0.068 | 0.72 | 0.35–1.47 | 0.365 |
| **heavy (**≥14 for men, ≥7 for women). | **0.72** | **0.54–0.96** | **0.027** | 0.43 | 0.16–1.16 | 0.095 |
| **Current smoking status** |  |  |  |  |  |  |
| no (never smoker, ex-smoker) | 1.00 |  |  | 1.00 |  |  |
| **current smoker** | **1.29** | **1.01–1.64** | **0.044** | 1.34 | 0.59–3.01 | 0.481 |
| **Perceived stress** |  |  |  |  |  |  |
| low (rare/mild) | 1.00 |  |  | 1.00 |  |  |
| **high (moderate/severe)** | **1.71** | **1.44–2.04** | **<0.001** | **1.75** | **1.11–2.75** | **0.016** |
| ^e^Chronic condition count |  |  |  |  |  |  |
| 0 | 1.00 |  |  | 1.00 |  |  |
| 1 | 0.97 | 0.79–1.19 | 0.768 | 1.32 | 0.53–3.26 | 0.549 |
| 2 | 0.96 | 0.77–1.20 | 0.738 | 1.49 | 0.58–3.83 | 0.408 |
| ≥3 | 1.31 | 0.94–1.83 | 0.113 | 1.47 | 0.47–4.64 | 0.507 |

Abbreviations: PV = Positional vertigo; abn = Abnormal; OR = Odds ratio; CI = Confidence interval; BMD = Bone mineral density; METs = Metabolic Equivalent Task-minutes.

^a^Abnormal BMD: lowest T-score of the total femur, femoral neck, and lumbar spine of less than −1.0.

^b^Sarcopenia: appendicular skeletal muscle mass index (ASM/height in meters squared) of <7.0 kg/m2 for men and <5.4 kg/m2 for women.

^c^Obesity: body fat mass percentage of ≥25% for men and ≥35% for women.

^d^MET: multiplying the days of physical activity per week by the duration (minutes) per day by a constant determined for each activity (vigorous activity: 8, moderate activity: 4, and walking: 3.3)

**^e^Chronic condition count** was categorized as 0, 1, 2, or ≥3 based on the total number of physician-diagnosed chronic diseases (e.g., hypertension, diabetes, cancer).

^abn^Romberg denoted failure on condition 4 (standing on compliant foam with eyes closed), indicating vestibular dysfunction.

Values were expressed in the form of ORs (95% CIs).

The control group was used as reference.

Bold values indicated statistical significance (*p* value <0.05).

**Supplementary Table 5. Sensitivity Analysis of Multinomial Logistic Regression for Current Positional Vertigo**

| **Variable** | **Current PV with *Romberg*** | | | **Current PV with *^abn^Romberg*** | | |
| --- | --- | --- | --- | --- | --- | --- |
|  | **OR** | **95% CI** | ***p* value** | **OR** | **95% CI** | ***p* value** |
| **Age** | **1.04** | **1.02–1.06** | **0.001** | **1.09** | **1.03–1.16** | **0.006** |
| **^a^Min T-score** | 1.02 | 0.83–1.24 | 0.882 | **0.49** | **0.25–0.99** | **0.046** |
| **Sex** |  |  |  |  |  |  |
| men | 1.00 |  |  | 1.00 |  |  |
| **women** | **2.14** | **1.27–3.61** | **0.004** | 2.95 | 0.83–10.52 | 0.095 |
| Household income |  |  |  |  |  |  |
| Q1 (low) | 1.44 | 0.90–2.33 | 0.130 | 0.51 | 0.18–1.43 | 0.200 |
| Q2 (lower-middle) | 0.78 | 0.47–1.29 | 0.328 | 0.37 | 0.10–1.35 | 0.132 |
| Q3 (upper-middle) | 0.72 | 0.42–1.25 | 0.245 | 0.26 | 0.05–1.32 | 0.104 |
| Q4 (higher) | 1.00 |  |  | 1.00 |  |  |
| **^b^Sarcopenia** |  |  |  |  |  |  |
| normal | 1.00 |  |  | 1.00 |  |  |
| **sarcopenia** | 1.03 | 0.72–1.47 | 0.885 | **2.97** | **1.08–8.12** | **0.035** |
| ^c^Obesity |  |  |  |  |  |  |
| normal | 1.00 |  |  | 1.00 |  |  |
| obesity | 0.92 | 0.67–1.26 | 0.598 | 1.46 | 0.45–4.75 | 0.530 |
| **Diagnosis of depression disorder** |  |  |  |  |  |  |
| no | 1.00 |  |  | 1.00 |  |  |
| **yes** | **2.18** | **1.26–3.77** | **0.005** | 1.18 | 0.31–4.44 | 0.807 |
| **Physical activity** |  |  |  |  |  |  |
| **low** (<600 ^d^METs/week) | **1.74** | **1.14–2.64** | **0.010** | **0.28** | **0.09–0.92** | **0.036** |
| moderate (600 – 2,999 METs/week) | 1.41 | 0.92–2.16 | 0.110 | 0.38 | 0.12–1.27 | 0.117 |
| vigorous (≥3,000 METs/week) | 1.00 |  |  | 1.00 |  |  |
| **Alcohol drinking** |  |  |  |  |  |  |
| none (<1 drink/week) | 1.00 |  |  | 1.00 |  |  |
| **moderate** (1–13 for men, 1–6 for women) | **0.63** | **0.40–0.98** | **0.039** | 0.37 | 0.10–1.42 | 0.149 |
| heavy **(**≥14 for men, ≥7 for women) | 0.68 | 0.38–1.21 | 0.186 | 0.70 | 0.12–4.04 | 0.687 |
| **Current smoking status** |  |  |  |  |  |  |
| no (never smoker, ex-smoker) | 1.00 |  |  | 1.00 |  |  |
| **current smoker** | 1.21 | 0.69–2.13 | 0.504 | 0.59 | 0.13–2.78 | 0.507 |
| **Perceived stress** |  |  |  |  |  |  |
| low (rare/mild) | 1.00 |  |  | 1.00 |  |  |
| **high (moderate/severe)** | **2.29** | **1.65–3.17** | **<0.001** | 1.95 | 0.86–4.42 | 0.107 |
| **^e^Chronic condition count** |  |  |  |  |  |  |
| 0 | 1.00 |  |  | 1.00 |  |  |
| 1 | 1.03 | 0.68–1.56 | 0.879 | 0.44 | 0.13–1.51 | 0.193 |
| 2 | 0.88 | 0.60–1.29 | 0.527 | 0.27 | 0.06–1.15 | 0.076 |
| **≥3** | **2.23** | **1.25–3.99** | **0.007** | 0.91 | 0.22–3.74 | 0.901 |

Abbreviations: PV = Positional vertigo; abn = Abnormal; OR = Odds ratio; CI = Confidence interval; METs = Metabolic Equivalent Task-minutes.

^a^Minimum T-score referred to the lowest value among the total femur, femoral neck, and lumbar spine T-scores

^b^Sarcopenia was defined as an appendicular skeletal muscle mass index (ASM/height in meters squared) of <7.0 kg/m2 for men and <5.4 kg/m2 for women.

^c^Obesity was defined as body fat mass percentage of ≥25% for men and ≥35% for women.

^d^MET: multiplying the days of physical activity per week by the duration (minutes) per day by a constant determined for each activity (vigorous activity: 8, moderate activity: 4, and walking: 3.3)

**^e^Chronic condition count** was categorized as 0, 1, 2, or ≥3 based on the total number of physician-diagnosed chronic diseases (e.g., hypertension, diabetes, cancer).

^abn^Romberg denoted failure on condition 4 (standing on compliant foam with eyes closed), indicating vestibular dysfunction.

Values were expressed as ORs with 95% CIs.

The control group was used as reference.

**As all participants in the ^abn^Romberg group had abnormal bone mineral density, bone health was assessed using the continuous variable minimum T-score.**

Bold values indicated significance (*p* value <0.05).

**Supplementary Table 6. Age-Stratified Sensitivity Analysis of Multinomial Logistic Regression by Age Group (< 65 years)**

| **Variable** | **Morning PV with Romberg** | | | **Morning PV with ^abn^Romberg** | | |
| --- | --- | --- | --- | --- | --- | --- |
|  | **OR** | **95% CI** | ***p* value** | **OR** | **95% CI** | ***p* value** |
| **Age** | **1.02** | **1.00–1.04** | **0.019** | **1.10** | **1.00–1.20** | **0.040** |
| Sex |  |  |  |  |  |  |
| men | 1.00 |  |  | 1.00 |  |  |
| **women** | **2.16** | **1.67–2.79** | **<0.001** | 2.21 | 0.69–7.06 | 0.180 |
| Household income |  |  |  |  |  |  |
| **Q1 (low)** | **1.62** | **1.17–2.24** | **0.004** | 0.81 | 0.23–2.86 | 0.740 |
| Q2 (lower-middle) | 1.12 | 0.84–1.51 | 0.437 | 1.08 | 0.30–3.91 | 0.908 |
| Q3 (upper-middle) | 1.12 | 0.86–1.44 | 0.408 | 0.83 | 0.21–3.28 | 0.789 |
| Q4 (higher) | 1.00 |  |  | 1.00 |  |  |
| ^a^Abnormal BMD |  |  |  |  |  |  |
| normal | 1.00 |  |  | 1.00 |  |  |
| abnormal BMD | 1.14 | 0.91–1.42 | 0.251 | 1.37 | 0.48–3.85 | 0.554 |
| ^b^Sarcopenia |  |  |  |  |  |  |
| normal | 1.00 |  |  | 1.00 |  |  |
| sarcopenia | 1.12 | 0.87–1.43 | 0.392 | 1.12 | 0.41–3.09 | 0.822 |
| ^c^Obesity |  |  |  |  |  |  |
| normal | 1.00 |  |  | 1.00 |  |  |
| obesity | 1.11 | 0.90–1.36 | 0.322 | 1.91 | 0.76–4.81 | 0.168 |
| Diagnosis of depression disorder |  |  |  |  |  |  |
| no | 1.00 |  |  | 1.00 |  |  |
| **yes** | **1.85** | **1.26–2.70** | **0.002** | **3.82** | **1.45–10.05** | **0.007** |
| Physical activity |  |  |  |  |  |  |
| low (<600 ^d^METs/week) | 1.05 | 0.81–1.36 | 0.702 | 0.91 | 0.24–3.39 | 0.883 |
| moderate (600 – 2,999 METs/week) | 1.09 | 0.86–1.38 | 0.459 | 0.72 | 0.25–2.04 | 0.533 |
| vigorous (≥3,000 METs/week) | 1.00 |  |  | 1.00 |  |  |
| Alcohol drinking |  |  |  |  |  |  |
| none (<1 drink/week) | 1.00 |  |  | 1.00 |  |  |
| moderate (1–13 for men, 1–6 for women) | 0.82 | 0.64–1.06 | 0.125 | 0.61 | 0.22–1.67 | 0.338 |
| **heavy (≥14 for men, ≥7 for women).** | **0.70** | **0.49–0.98** | **0.038** | 0.67 | 0.14–3.17 | 0.610 |
| Current smoking status |  |  |  |  |  |  |
| no (never smoker, ex-smoker) | 1.00 |  |  | 1.00 |  |  |
| **current smoker** | **1.43** | **1.08–1.90** | **0.013** | 0.64 | 0.17–2.36 | 0.503 |
| Perceived stress |  |  |  |  |  |  |
| low (rare/mild) | 1.00 |  |  | 1.00 |  |  |
| **high (moderate/severe)** | **1.68** | **1.37–2.06** | **<0.001** | 1.84 | 0.80–4.25 | 0.150 |
| ^e^Chronic condition count |  |  |  |  |  |  |
| 0 | 1.00 |  |  | 1.00 |  |  |
| 1 | 0.96 | 0.76–1.21 | 0.703 | 1.52 | 0.40–5.72 | 0.534 |
| 2 | 0.89 | 0.68–1.16 | 0.385 | 1.16 | 0.26–5.17 | 0.843 |
| ≥3 | 1.40 | 0.91–2.16 | 0.124 | 0.20 | 0.02–2.09 | 0.180 |

Abbreviations: PV = Positional vertigo; abn = Abnormal; OR = Odds ratio; CI = Confidence interval; BMD = Bone mineral density; METs = Metabolic Equivalent Task-minutes.

Sample size: total (n=6,200), Morning PV with Romberg (n = 736), Morning PV with ^abn^Romberg (n = 28), control (n = 5,436).

This analysis was conducted on individuals aged 40-64 years.

^a^Abnormal BMD: lowest T-score of the total femur, femoral neck, and lumbar spine of less than −1.0.

^b^Sarcopenia: appendicular skeletal muscle mass index (ASM/height in meters squared) of <7.0 kg/m2 for men and <5.4 kg/m2 for women.

^c^Obesity: body fat mass percentage of ≥25% for men and ≥35% for women.

^d^MET: multiplying the days of physical activity per week by the duration (minutes) per day by a constant determined for each activity (vigorous activity: 8, moderate activity: 4, and walking: 3.3)

**^e^Chronic condition count** was categorized as 0, 1, 2, or ≥3 based on the total number of physician-diagnosed chronic diseases (e.g., hypertension, diabetes, cancer).

^abn^Romberg denoted failure on condition 4 (standing on compliant foam with eyes closed), indicating vestibular dysfunction.

Values were expressed as ORs with 95% CIs.

The control group was used as reference.

Bold values indicated statistical significance (*p* value <0.05).

**Supplementary Table 7. Age-Stratified Sensitivity Analysis of Multinomial Logistic Regression by Age Group (≥ 65 years)**

| **Variable** | **Morning PV with Romberg** | | | **Morning PV with ^abn^Romberg** | | |
| --- | --- | --- | --- | --- | --- | --- |
|  | **OR** | **95% CI** | ***p* value** | **OR** | **95% CI** | ***p* value** |
| **Age** | 1.02 | 0.99–1.05 | 0.243 | **1.09** | **1.01–1.18** | **0.031** |
| Sex |  |  |  |  |  |  |
| men | 1.00 |  |  | 1.00 |  |  |
| **women** | **1.64** | **1.19–2.24** | **0.002** | **2.09** | **1.00–4.38** | **0.050** |
| Household income |  |  |  |  |  |  |
| Q1 (low) | 1.31 | 0.79–2.17 | 0.301 | 1.81 | 0.62–5.26 | 0.277 |
| Q2 (lower-middle) | 1.23 | 0.72–2.10 | 0.439 | 1.26 | 0.32–5.01 | 0.742 |
| Q3 (upper-middle) | 1.01 | 0.54–1.89 | 0.977 | 1.42 | 0.33–6.16 | 0.637 |
| Q4 (higher) | 1.00 |  |  | 1.00 |  |  |
| ^a^Abnormal BMD |  |  |  |  |  |  |
| normal | 1.00 |  |  | 1.00 |  |  |
| abnormal BMD | 1.17 | 0.80–1.71 | 0.418 | 3.30 | 0.83–13.07 | 0.089 |
| **^b^Sarcopenia** |  |  |  |  |  |  |
| normal | 1.00 |  |  | 1.00 |  |  |
| **sarcopenia** | 1.27 | 0.97–1.66 | 0.085 | **2.48** | **1.28–4.83** | **0.007** |
| ^c^Obesity |  |  |  |  |  |  |
| normal | 1.00 |  |  | 1.00 |  |  |
| obesity | 0.85 | 0.65–1.10 | 0.213 | 0.85 | 0.38–1.87 | 0.678 |
| Diagnosis of depression disorder |  |  |  |  |  |  |
| no | 1.00 |  |  | 1.00 |  |  |
| yes | 0.86 | 0.48–1.55 | 0.622 | 2.02 | 0.83–4.94 | 0.123 |
| Physical activity |  |  |  |  |  |  |
| low (<600 ^d^METs/week) | 1.34 | 0.94–1.92 | 0.105 | 1.24 | 0.53–2.94 | 0.619 |
| **moderate (600 – 2,999 METs/week)** | **1.39** | **1.02–1.90** | **0.036** | 0.79 | 0.43–1.48 | 0.465 |
| vigorous (≥3,000 METs/week) | 1.00 |  |  | 1.00 |  |  |
| Alcohol drinking |  |  |  |  |  |  |
| none (<1 drink/week) | 1.00 |  |  | 1.00 |  |  |
| moderate (1–13 for men, 1–6 for women) | 0.82 | 0.57–1.18 | 0.278 | 0.88 | 0.39–1.97 | 0.750 |
| heavy (≥14 for men, ≥7 for women). | 0.89 | 0.55–1.44 | 0.632 | 0.27 | 0.07–1.10 | 0.067 |
| Current smoking status |  |  |  |  |  |  |
| no (never smoker, ex-smoker) | 1.00 |  |  | 1.00 |  |  |
| current smoker | 1.01 | 0.68–1.50 | 0.945 | 1.80 | 0.72–4.47 | 0.208 |
| Perceived stress |  |  |  |  |  |  |
| low (rare/mild) | 1.00 |  |  | 1.00 |  |  |
| **high (moderate/severe)** | **1.88** | **1.36–2.60** | **<0.001** | **1.83** | **1.01–3.33** | **0.048** |
| ^e^Chronic condition count |  |  |  |  |  |  |
| 0 | 1.00 |  |  | 1.00 |  |  |
| 1 | 1.10 | 0.73–1.68 | 0.639 | 1.18 | 0.43–3.29 | 0.746 |
| 2 | 1.28 | 0.81–2.01 | 0.292 | 1.88 | 0.59–5.94 | 0.283 |
| ≥3 | 1.36 | 0.83–2.21 | 0.219 | 2.26 | 0.61–8.44 | 0.223 |

Abbreviations: PV = Positional vertigo; abn = Abnormal; OR = Odds ratio; CI = Confidence interval; BMD = Bone mineral density; METs = Metabolic Equivalent Task-minutes.

Sample size: total (n = 2,312), Morning PV with Romberg (n = 456), Morning PV with ^abn^Romberg (n = 63), Control (n = 1,793).

This analysis was conducted on individuals aged ≥65 years.

^a^Abnormal BMD: lowest T-score of the total femur, femoral neck, and lumbar spine of less than −1.0.

^b^Sarcopenia: appendicular skeletal muscle mass index (ASM/height in meters squared) of <7.0 kg/m^2^ for men and <5.4 kg/m^2^ for women.

^c^Obesity: body fat mass percentage of ≥25% for men and ≥35% for women.

^d^MET: multiplying the days of physical activity per week by the duration (minutes) per day by a constant determined for each activity (vigorous activity: 8, moderate activity: 4, and walking: 3.3)

**^e^Chronic condition count** was categorized as 0, 1, 2, or ≥3 based on the total number of physician-diagnosed chronic diseases (e.g., hypertension, diabetes, cancer).

^abn^Romberg denoted failure on condition 4 (standing on compliant foam with eyes closed), indicating vestibular dysfunction.

Values were expressed as ORs with 95% CIs.

The control group was used as a reference.

Bold values indicated statistical significance (*p* value <0.05).
